# Neuromuscular Adaptations to Same Versus Separate Muscle‐Group Concurrent Aerobic and Strength Training in Recreationally Active Males and Females

**DOI:** 10.1111/sms.70025

**Published:** 2025-02-08

**Authors:** Joshua F. Feuerbacher, Mats W. Jacobs, Paulina Heumann, Fernando Pareja‐Blanco, Anthony C. Hackney, Jonas Zacher, Moritz Schumann

**Affiliations:** ^1^ Department of Sports Medicine and Exercise Therapy University of Technology Chemnitz Germany; ^2^ Department of Molecular and Cellular Sports Medicine German Sport University Cologne Germany; ^3^ Faculty of Sports Sciences, Physical Performance & Sports Research Center Universidad Pablo de Olavide Seville Spain; ^4^ University of North Carolina Chapel Hill North Carolina USA; ^5^ Institute of Cardiology and Sports Medicine German Sports University Cologne Cologne Germany

**Keywords:** interference effect, muscle hypertrophy, one‐repetition maximum, rapid force development

## Abstract

Combining aerobic and strength training may attenuate neuromuscular adaptations, particularly when both target the same muscle group. This study assessed whether separating the training modalities by muscle groups mitigates this interference. Ninety‐six participants (56 males and 40 females) completed a 12‐week intervention, divided into three groups: (1) LHLS (lower‐body high‐intensity interval (HIIT) and strength training), (2) LHUS (lower‐body HIIT and upper‐body strength training), and (3) LSUS (lower‐ and upper‐body strength training). Maximal (1RM) and explosive strength were assessed using load–velocity profiling, with mean propulsive velocity (MPV) at 30%, 50%, 70%, and 90% of 1RM as a measure of explosive strength. Muscle cross‐sectional area (CSA) of the M. vastus lateralis and M. pectoralis major was measured using panoramic ultrasound. Lower‐body adaptations were compared between LHLS and LSUS, and upper‐body adaptations were compared between LHUS and LSUS. MPV at 70% and 90% of 1RM for the squat (LHLS and LSUS) and bench press (LHUS and LSUS) showed improvements (*p* < 0.050), with no significant between‐group differences. Squat 1RM improved in both LHLS and LSUS, and bench press 1RM increased in both LHUS and LSUS (all *p* < 0.001). M. vastus lateralis CSA increased in LHLS (*p* = 0.029) but not in LSUS, whereas M. pectoralis major CSA increased in both LHUS and LSUS (*p* < 0.001), with no between‐group differences. No sex‐based differences were observed. Concurrent aerobic and strength training does not impair explosive strength, maximal strength, or muscle hypertrophy, regardless of whether the same or separate muscle groups are targeted.

## 1 Introduction

Several studies have shown that combining aerobic and strength training may impair rapid force production in both males [1] and females [2, 3], even when the aerobic training volume is low. However, this interference in explosive strength development has been demonstrated exclusively in studies where strength and aerobic training were performed within the same muscle group [4, 5], while evidence is lacking with regard to aerobic and strength training being performed by separate muscle groups. Importantly, training for both aerobic and strength development within the same session is often unavoidable, and thus, separating aerobic and strength training by muscle group may help to reduce potential interference effects, although this has primarily been shown for maximal strength and muscle size [6], rather than explosive strength.

Explosive strength, in particular, is influenced by neural factors involving coordinated interactions between agonists, antagonists, and synergists, along with a high discharge rate of motor units at the onset of contraction [7]. This is important as a previous work has shown that explosive strength adaptations may be compromised due to neural maladaptation when aerobic and strength training are performed concurrently [1]. In addition to neural factors, other factors, such as muscle architecture [7] and tendon properties [8], significantly impact explosive strength, and evidence suggests that high‐intensity interval (HIIT) cycling performed after heavy‐resistance exercise may decrease rate of force development (RFD), potentially due to muscle architectural changes [9]. Taken together, it may be speculated that the potential for interference in explosive strength development appears to be a local phenomenon, occurring primarily when aerobic and strength training target the same muscle group.

In line with this, we previously showed that lower‐body HIIT in both males and females leads to acute reductions in lower‐body explosive strength performance, while upper‐body explosive strength was maintained [10, 11]. However, whether prolonged concurrent aerobic and strength training compromises explosive strength adaptations in the upper body remains unclear. Given that maximal strength and muscle mass affect explosive strength as well [7], gaining further insights into neuromuscular adaptations is warranted and could help refine training approaches for diverse populations, particularly as explosive strength is essential for both athletic performance [12] and overall health [13, 14].

Moreover, a notable limitation of the current evidence is the underrepresentation of studies involving females, as most studies are performed with male participants. While it is suggested that females may experience different adaptations than males due to distinct physiological, hormonal, and morphological characteristics [15], the lack of studies including female participants raises concerns about the generalizability of previous findings derived from male participants. This is particularly important, as the potential influence of menstrual phases in female participants of reproductive age has not been adequately addressed [16].

Thus, the primary aim of this study was to assess whether separating aerobic and strength training by muscle group mitigates the interference effect on explosive strength compared to concurrent training of the same muscle group in recreationally active males and females. Moreover, we aimed to evaluate whether potential interference is sex‐specific. Our secondary aim was to evaluate the impact of separating aerobic and strength training by muscle group on maximal strength and muscle mass. We hypothesized that separating aerobic and strength training in different muscle groups would result in similar increases in explosive strength, maximal strength, and muscle mass compared to sole strength training. Conversely, when aerobic and strength training target the same muscle group, the development of explosive strength would be attenuated. Moreover, we hypothesize that no sex‐specific differences will occur in the development of explosive strength, maximal strength, or muscle mass.

## 2 Methods

### 2.1 Participants

The study included both male and premenopausal female participants. Participants were eligible if they met the following criteria: (1) nonsmokers, (2) free from chronic or acute injuries, (3) aged 18–40 years, and (4) physically active. Specifically, eligibility criteria included aerobic fitness [17] (peak oxygen uptake (VO_2peak_): 35–50 mL·kg^−1^·min^−1^ for males, 30–45 mL·kg^−1^·min^−1^ for females) and strength capacity [18] (squat one‐repetition maximum [1RM]: 0.8–1.2 kg·kg^−1^ body mass [BM] for males, 0.6–1.0 kg·kg^−1^ for females; bench press 1RM: 0.6–1.0 kg·kg^−1^ for males, 0.4–0.6 kg·kg^−1^ for females). Female participants were eligible if they had a natural menstrual cycle with regular menstrual bleeding for at least 3 consecutive months before the study and had not used hormonal contraceptives for 1.5 years before participation [19]. Information regarding menstrual cycles and monthly bleeding was collected during a familiarization interview.

**FIGURE 1:**
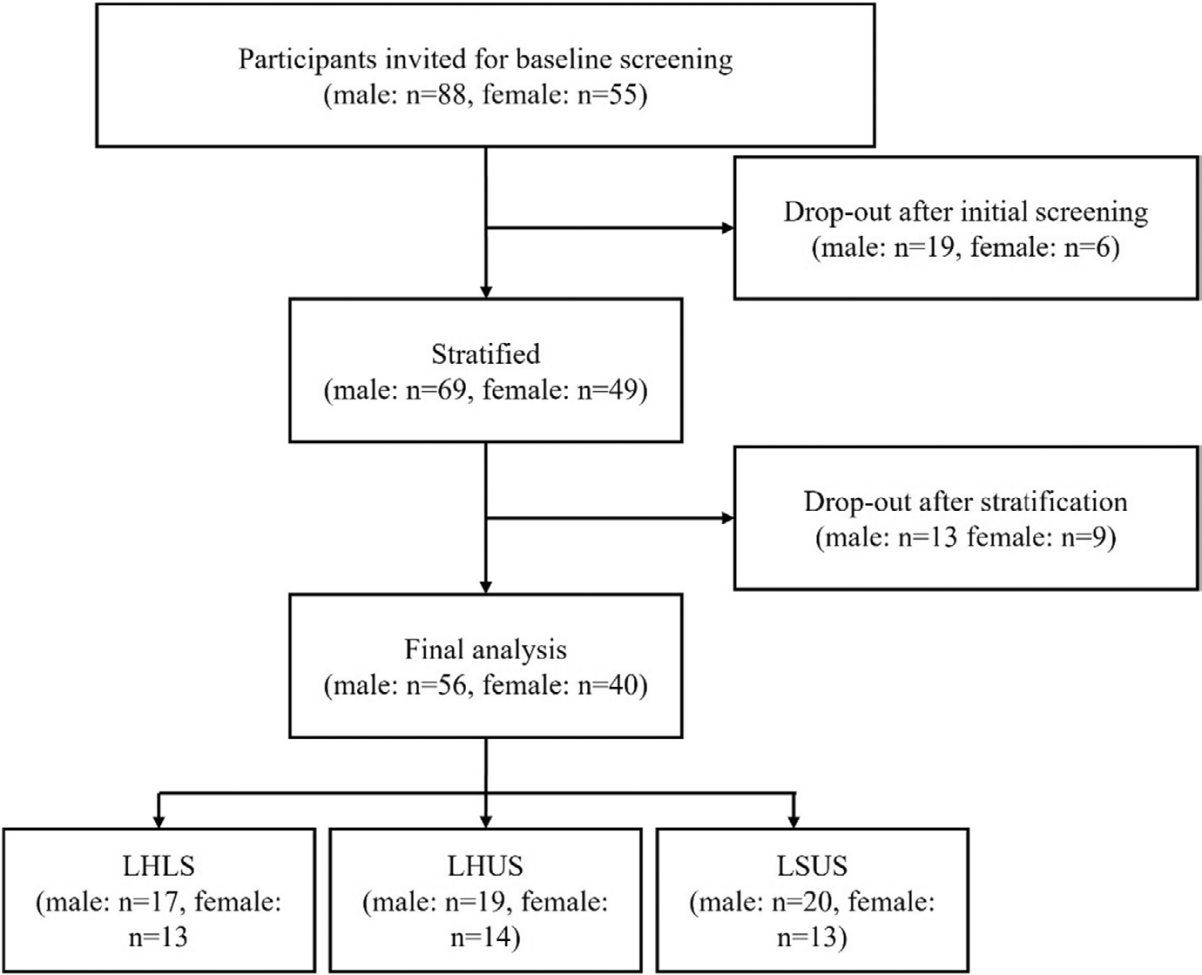
Flowchart of the recruitment process. LHLS, lower‐body high‐intensity interval training (HIIT) and lower‐body strength; LHUS, lower‐body HIIT and upper‐body strength; LSUS, lower‐body and upper‐body strength.

After initial applications for study participation, 88 males and 55 females were invited to the baseline screening. Following verifying the aerobic and strength performance characteristics, 69 males and 49 females were enrolled in the study and commenced with the training intervention (Figure 1). However, 13 males and 9 females dropped out due to reasons unrelated to the study, including injury (male: 2, female: 1), prolonged illness/health issues (male: 1, female: 2), personal reasons (male: 1, female: 2), COVID‐19 pandemic (male: 6), time constraints (male:1, female: 3), and pregnancy (female: 1). Finally, 56 males (age: 28.7 ± 5.5 years, height: 182.6 ± 6.8 cm, BM: 80.1 ± 8.7 kg) and 40 females (age: 26.9 ± 6.0 years, height: 168.9 ± 8.0 cm, BM: 65.2 ± 10.3 kg) were included in the final analysis. Prior to inclusion, all participants received instructions regarding potential risks and provided written informed consent. This study received approval from the local institutional board and adhered to the principles of the Declaration of Helsinki (German Sport University, 036/2019).

### 2.2 Experimental Design

The participants underwent a 12‐week training period and were stratified into three groups: Group 1 (LHLS: *n* = 17 males, *n* = 13 females) performed lower‐body HIIT (LH) combined with lower‐body strength training (LS). Group 2 (LHUS: *n* = 19 males, *n* = 14 females) performed aerobic and strength training separated by muscle group, that is, lower‐body HIIT in combination with upper‐body strength training (US). The third group (LSUS: *n* = 20 males, *n* = 13 females) served as a control group and performed lower‐ and upper‐body strength training.

Participants were first familiarized with study procedures before undergoing baseline testing, which included assessments of aerobic fitness, strength capacity, and muscle cross‐sectional area (CSA). After baseline testing, they were stratified into experimental or control groups based on squat and bench press maximal strength, aerobic capacity (VO_2peak_, peak power output [PPO]), and menstrual cycle length for females (LHLS = 28.6 ± 0.8; LHUS = 27.6 ± 3.5; LSUS = 29.5 ± 2.8 days). The intervention was divided into three 4‐week training blocks, with strength testing conducted at the end of each block, while muscle CSA was assessed postintervention.

In contrast to men, the testing and training in females were aligned with their menstrual cycle, as previous research suggests that ovarian hormones may influence aerobic and strength performance [20]. Thus, females trained for three menstrual cycles instead of 12 weeks. Baseline aerobic performance was assessed during the luteal phase before the intervention, while strength and CSA assessments were always conducted during the follicular phase (between Days 3 and 7 of menstruation). Similar to men, strength testing was conducted at the end of each cycle while muscle CSA was assessed postintervention.

### 2.3 Measurements

#### 2.3.1 Aerobic Testing

To assess the basal PPO, participants completed a graded exercise test on an electronically braked cycle ergometer (Ergoline, Ergoline GmbH, Bitz, Germany) until they reached volitional exhaustion. Before the test, BM was measured using a Sanitas system (Hans Dinslage GmbH, Uttenweiler, Germany). The test commenced with a 5‐min warm‐up at 80 W for males and 50 W for females. Subsequently, the resistance was incremented every 3 min by 30 W. Gas exchange was analyzed using a Metalyzer 3B, which was calibrated according to the manufacturer's recommendations before each test (Cortex, Leipzig, Germany). The V̇O_2peak_ was determined as the highest 30‐s average and was utilized to quantify the level of aerobic fitness.

#### 2.3.2 Strength Testing

Baseline strength performance was assessed through an incremental 1RM test in the squat and bench press exercises, with participants always completing the squat first. Alongside the mean propulsive velocity (MPV), the eccentric displacement was obtained during each repetition using a linear velocity transducer (T‐Force System, Ergotech Consulting, Murcia, Spain). Strength capacity was evaluated using load–velocity profiles during squat and bench press, from which the 1RM and the MPV attained against different submaximal loads were derived as strength indicators. The linear velocity transducer recorded the MPV and eccentric displacement during each repetition. The propulsive phase was defined as the part of the concentric movement where the measured acceleration exceeded gravitational acceleration [21]. Velocity was sampled at a frequency of 1000 Hz and processed using custom software (T‐Force Dynamic Measurement System, version 2.3).

The test began with a 5‐min individualized warm‐up on a stationary cycle ergometer. Before the squat exercise, participants performed 10 bodyweight squats followed by 3–6 repetitions using the Smith machine barbell (22 kg) as an exercise‐specific warm‐up. Depth and foot positioning were recorded to ensure consistency across all sets. For the bench press exercise, participants completed 10 push‐ups and 3–6 repetitions with the unloaded barbell as a warm‐up, with grip width recorded for consistency. Prior to each set, participants were instructed to maintain a controlled eccentric phase until the reversal point, hold the position for 1.5 s, and then perform the concentric phase with maximal velocity in a nonballistic manner. For both the squat and bench press exercises, the test commenced with an initial load of 22 kg and was progressively increased until participants could no longer lift the weight with the correct technique. Load increments and the number of repetitions were adjusted based on the velocity achieved during each set. Three, two, and one repetitions were performed for high (MPV > 1.0 m·s^−1^), moderate (0.75 m·s^−1^ ≤ MPV ≤ 1.0 m·s^−1^), and low velocities (< 0.75 m·s^−1^), respectively. Rest between sets was set at 2 min. Following the test, individual load–velocity relationships were calculated using linear regression to assess MPV at 30%, 50%, 70%, and 90% of the 1RM. Subsequently, absolute movement velocities at Week‐0 loads were compared to display changes in explosive strength.

#### 2.3.3 Muscle Cross‐Sectional Area Assessment

To assess lower‐ and upper‐body hypertrophy, the CSA of the M. vastus lateralis and M. pectoralis major was assessed using panoramic ultrasound imaging (VividTM iq Premium, GE Healthcare, Chicago IL, the United States). Three panoramic CSA images were taken at 50% of the femur length (lateral aspect of the distal diaphysis to the greater trochanter) for the M. vastus lateralis, and for the M. pectoralis major, at the midpoint of a perpendicular line extending from the clavicle. Participants were positioned in a supine position while five panoramic images per extremity were captured using the 9 L‐RS linear array transducer (GE Healthcare, Chicago IL, the United States). One trained rater conducted the assessment of CSA. For each muscle group, three images were analyzed using ImageJ Software (version 1.53 t, 2022, National Institutes of Health, Bethesda, MD). Following the methodology of previous studies [22, 23], the mean of the two closest measured cross sections was used for statistical analyses. Due to the female anatomy considerations, the M. pectoralis major assessment was only performed in males.

#### 2.3.4 Training

The training period lasted for 12 weeks in males and 3 menstrual cycles in females (12.3 ± 0.7 weeks). For LHLS and LHUS, aerobic and strength training were performed within a single session in an alternating order and within approximately 5–10 min. Similarly, for the LSUS group lower‐body and upper‐body strength exercises were performed in the same session and in alternating order. Overall training session duration was the same for all three groups (~1.5 h). The training program was structured to align with recommendations for physically active individuals [17]. The primary goal was to enhance both aerobic capacity and strength through a periodized approach, incorporating aerobic sessions at high intensities [24], along with hypertrophy, maximal, and explosive strength‐focused strength training protocols [1, 25]. All training sessions were supervised by trained personnel.

##### 2.3.4.1 Aerobic Training

The HIIT protocol was designed using intermittent aerobic intervals, consisting of 4 min of high‐intensity exercise followed by 3 min of active recovery at a lower load. The training intensity of the intervals increased from 70% PPO in the first block to 80% PPO in the last block, corresponding to 80%–90% of VO_2peak_ as determined by pilot testing. Recovery intensity between intervals was consistently set at 40% PPO. The warm‐up program before each aerobic training consisted of 4 min of low‐intensity cycling at 40% PPO.

##### 2.3.4.2 Strength Training

The strength training consisted of three mesocycles, each lasting 4 weeks, consisting of hypertrophy, maximal strength, and explosive strength, respectively. Overall, a total of 26 training sessions were performed. Strength exercises comprised of squat, leg press, leg curl, and knee extension for the lower body, and bench press, incline bench press, seated rowing, and elbow extension for the upper body. The main exercises (squat, leg press, bench press, and incline bench press) aimed to enhance performance specifically tested in incremental 1RM tests, while the supplemental exercises (leg curl, knee extension, seated rowing, and elbow extension) were intended to prevent imbalances throughout the training period.

A detailed summary of the exercises and the respective loads can be found in Table S1 of Data S1. Briefly, during the first mesocycle, the training goal was to increase muscle mass, with exercises performed in three sets of eight to ten repetitions at a load of 75%–80% of 1RM. The second mesocycle focused on increasing maximal strength, involving three sets of three to five repetitions at a load of 85%–90% of 1RM for each exercise. In the last mesocycle, the main exercises were executed in three sets of six repetitions at a low load of 45%–50% of 1RM mixed with three sets of three repetitions at 90%–95% of 1RM. Rest between sets was 2 min, and between exercises, 5 min. Participants were instructed to perform each repetition with maximal concentric velocity. Within the mesocycles, there was a progressive adjustment of training loads based on the participants' training progress (approximately 2.5% per week).

### 2.4 Statistical Analyses

Data are presented as mean ± standard deviation. Statistical analyses were performed in R and RStudio (version 2024.04.0+753) using the “rstatix” package. The normality of distribution was assessed via the Shapiro–Wilk test, supplemented by visual inspection using Q‐Q plots. All data met the assumptions of normality.

To examine whether combined aerobic and strength training targeting the same muscle groups impacts explosive strength (i.e., MPV), maximal strength (i.e., 1RM), and muscle CSA, we compared lower‐body maximal strength and explosive strength in the squat exercise, and the CSA of the vastus lateralis between the LHLS and LSUS groups. Additionally, to assess whether concurrent aerobic and strength training targeting separate muscle groups impacts 1RM, MPV, and muscle CSA, we compared upper‐body maximal strength and explosive strength in the bench press exercise and the CSA of the pectoralis major between the LHUS and LSUS groups. A 2 × 4 repeated‐measures ANOVA (2 groups, 4 time points: preintervention, Week 4, Week 8, and postintervention) was used to analyze changes in maximal and explosive strength. For muscle CSA, we used a 2 × 2 repeated‐measures ANOVA (2 groups, 2 time points: preintervention and postintervention). Additionally, we employed a 2 × 4 × 2 MANOVA (2 groups, 4 time points, 2 sexes) to assess the interaction effects of training interventions and sex on explosive and maximal strength, and a 2 × 2 × 2 MANOVA (2 groups, 2 time points, 2 sexes) for changes in muscle CSA, where sex was treated as a potential factor.

When sphericity was violated, the Greenhouse–Geisser correction was used to adjust the degrees of freedom. *P*‐values for pairwise comparisons were post hoc corrected using the Bonferroni method. Additionally, the effect size was determined using partial eta squared (*ƞ* ^2^). A value of 0.01 corresponds to a small effect, a value of 0.06 to a medium effect, and a value of 0.14 to a large effect [26].

## 3 Results

The corresponding *F* statistics to the performed analyses are available in the results section of Data S1. Females completed 93.8% ± 2.8% (LHLS: 94.8% ± 2.3%, LHUS: 91.3% ± 1.6%, LSUS: 94.1% ± 1.0%) of their training sessions, while adherence for males was 95.7% ± 4.3% (LHLS: 95.2% ± 3.1%, LHUS: 96.4% ± 3.3%, LSUS: 94.7% ± 3.0%). The average menstrual cycle length for the included female participants was 28.6 ± 2.7 days. Table 1 presents the anthropometric and performance characteristics, grouped by sex. No significant differences were observed between groups at baseline (*p* > 0.050). Furthermore, our analysis indicated that sex did not affect the training adaptations for explosive strength, maximal strength, and muscle CSA (all, *p* > 0.050).

**TABLE 1:** Anthropometric and performance characteristics of the participants clustered by sex.

Group	Sex	Age [years]	Height [cm]	Body mass [kg]	VO_2_peak [mL min^−1^ kg^−1^]	PPO [Watt]	Relative PPO [Watt kg^−1^]	Squat 1RM [kg]	Relative squat 1RM [kg kg^−1^]	Bench press 1RM [kg]	Relative bench press 1RM [kg kg^−1^]
LHLS (*n* = 17)	Male (*n* = 56)	28.2 ± 6.1	181.4 ± 6.5	78.1 ± 8.7	44.9 ± 6.9	268.7 ± 39.4	3.47 ± 0.58	87.4 ± 17.9	1.13 ± 0.26	66.2 ± 13.4	0.86 ± 0.18
LHUS (*n* = 19)		28.8 ± 5.1	183.1 ± 6.9	82.5 ± 8.4	43.6 ± 4.3	286.1 ± 42.7	3.48 ± 0.50	93.1 ± 20.7	1.14 ± 0.24	66.2 ± 8.6	0.80 ± 0.11
LSUS (*n* = 20)		29.1 ± 5.6	183.3 ± 7.0	79.5 ± 8.9	44.6 ± 6.9	270.8 ± 49.1	3.42 ± 0.57	94.8 ± 19.1	1.20 ± 0.25	68.4 ± 13.9	0.86 ± 0.15
Total		**28.7 ± 5.5**	**182.6 ± 6.8**	**80.1 ± 8.7**	**44.4 ± 6.1**	**275.3 ± 44.1**	**3.45 ± 0.54**	**91.9 ± 19.2**	**1.16 ± 0.25**	**67.0 ± 12.0**	**0.84 ± 0.15**
LHLS (*n* = 13)	Female (*n* = 40)	27.8 ± 6.1	168.5 ± 7.3	64.7 ± 8.7	37.9 ± 2.5	178.3 ± 28.9	2.76 ± 0.30	59.9 ± 12.0	0.93 ± 0.15	34.8 ± 7.7	0.55 ± 0.11
LHUS (*n* = 14)		26.4 ± 5.6	169.9 ± 9.2	67.3 ± 11.2	39.9 ± 4.5	194.8 ± 36.0	2.92 ± 0.42	62.9 ± 11.3	0.95 ± 0.19	35.4 ± 6.0	0.53 ± 0.08
LSUS (*n* = 13)		26.6 ± 6.8	168.2 ± 7.8	63.4 ± 11.1	39.9 ± 4.8	192.7 ± 31.6	3.07 ± 0.48	64.7 ± 11.6	1.05 ± 0.22	38.5 ± 8.0	0.60 ± 0.13
Total		26.9 ± 6.0	168.9 ± 8.0	65.2 ± 10.3	39.3 ± 4.1	188.7 ± 32.0	2.92 ± 0.42	62.5 ± 11.5	0.97 ± 0.19	36.2 ± 7.3	0.56 ± 0.11

Abbreviations: 1RM, one‐repetition maximum; LHLS, lower‐body high‐intensity interval training (HIIT) and lower‐body strength; LHUS, lower‐body HIIT and upper‐body strength; LSUS, lower‐body and upper‐body strength; PPO, peak power output; VO_2_peak, maximal oxygen consumption.

### 3.1 Explosive Strength

#### 3.1.1 Same Muscle‐Group Concurrent Aerobic and Strength Training

**FIGURE 2:**
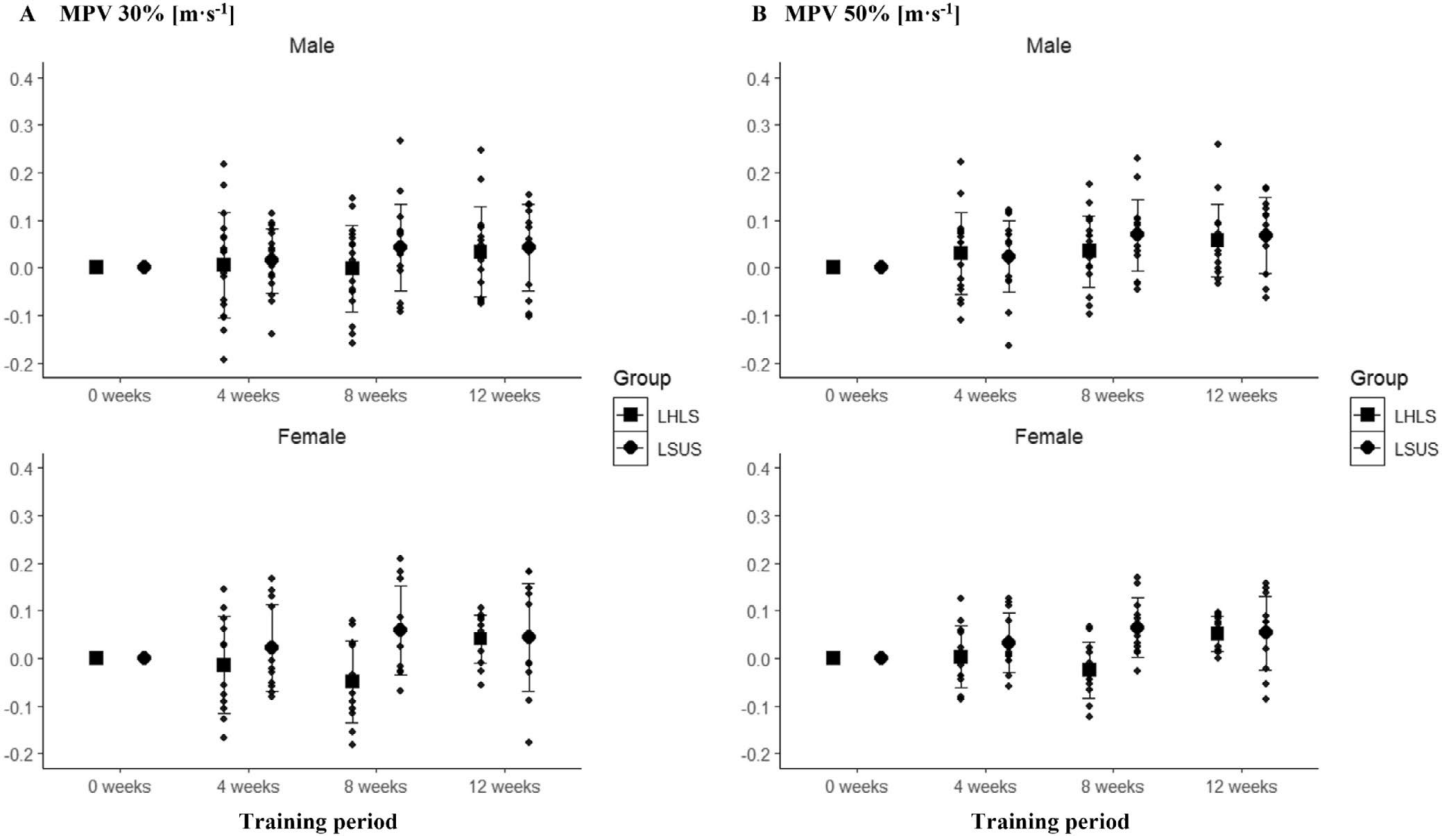
(A) Absolute change in MPV in the squat for LHLS and LSUS over the 12‐week training period at 30% of the Week‐0 1RM clustered by sex. (B) Absolute change in MPV in the squat for LHLS and LSUS over the 12‐week training period at 50% of the Week‐0 1RM clustered by sex. LHLS, lower‐body high‐intensity interval training and lower‐body strength; LSUS, lower‐body and upper‐body strength; MPV, mean propulsive velocity.

The corresponding mean changes and standard deviations are provided in Table S2 of Data S1. At 30% and 50% of 1RM, no significant main effect for time was observed in males (30%: *p* = 0.187; 50%: *p* = 0.020) or females (30%: *p* = 0.502; 50%: *p* = 0.132). Additionally, there was no significant interaction between time and group in either males (30%: *p* = 0.790; 50%: *p* = 0.790) or females (30%: *p* = 0.374; 50%: *p* = 0.244). Although a significant main effect for time was found at 50% in males, pairwise comparisons revealed no statistically significant differences between time points (*p* > 0.050) (Figure 2A,B).

**FIGURE 3:**
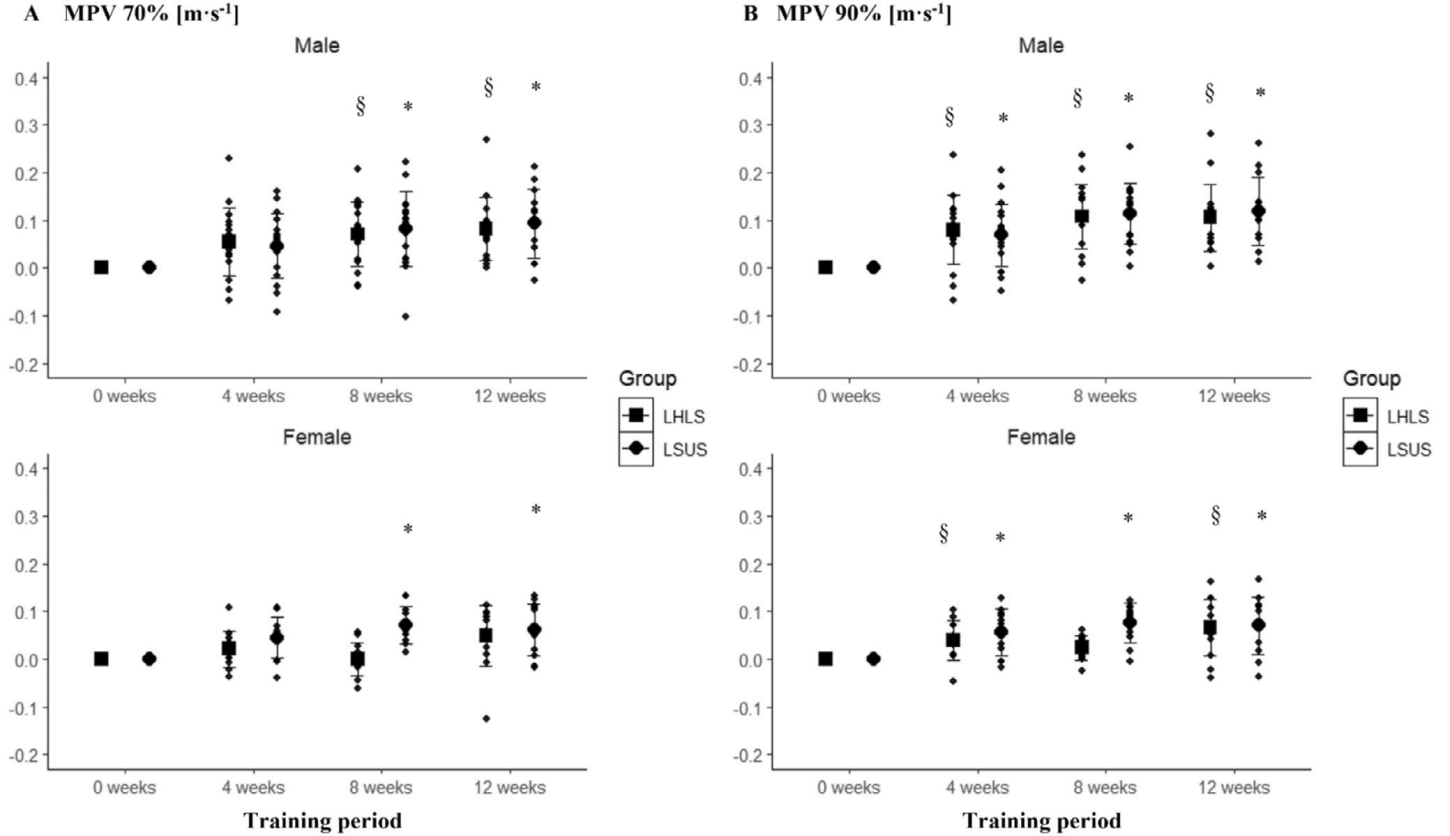
(A) Absolute change in MPV in the squat for LHLS and LSUS over the 12‐week training period at 70% of the Week‐0 1RM clustered by sex. (B) Absolute change in MPV in the squat for LHLS and LSUS over the 12‐week training period at 90% of the Week‐0 1RM clustered by sex. *Indicates significant changes compared to pretraining intervention in LSUS. ^§^Indicates significant changes compared to pretraining intervention in LHLS. LHLS, lower‐body high‐intensity interval training and lower‐body strength; LSUS, lower‐body and upper‐body strength; MPV, mean propulsive velocity.

Significant main effects for time were observed at 70% of 1RM in both males (*p* < 0.001) and females (*p* < 0.001), but no statistically significant interaction between time and group was observed (males: *p* = 0.768, females: *p* = 0.083). Statistically significant differences were present between preintervention and postintervention in LHLS for males (13.7% ± 11.8%; *p* < 0.001) but not in females (9.7% ± 11.9%; *p* = 0.112) and in LSUS (males: 15.3% ± 11.7%; *p* < 0.001; females: 14.7% ± 15.7%; *p* < 0.023) (Figure 3A). At 90% of 1RM, a statistically significant main effect for time was found in males (*p* < 0.001) and females (*p* < 0.001), with a statistically significant interaction between group and time in females (*p* = 0.024) but not in males (*p* = 0.728). Pairwise comparisons showed statistically significant differences between preintervention and postintervention in LHLS (males: 26.2% ± 19.6%; *p* < 0.001; females: 18.1% ± 18.6%; *p* = 0.010) and LSUS (males: 28.9% ± 18.2%; *p* < 0.001; females: 20.3% ± 17.3%; *p* = 0.011) (Figure 3B). However, no statistically significant differences between the groups were present in females at any time point (*p* > 0.050).

#### 3.1.2 Separate Muscle‐Group Concurrent Aerobic and Strength Training

**FIGURE 4:**
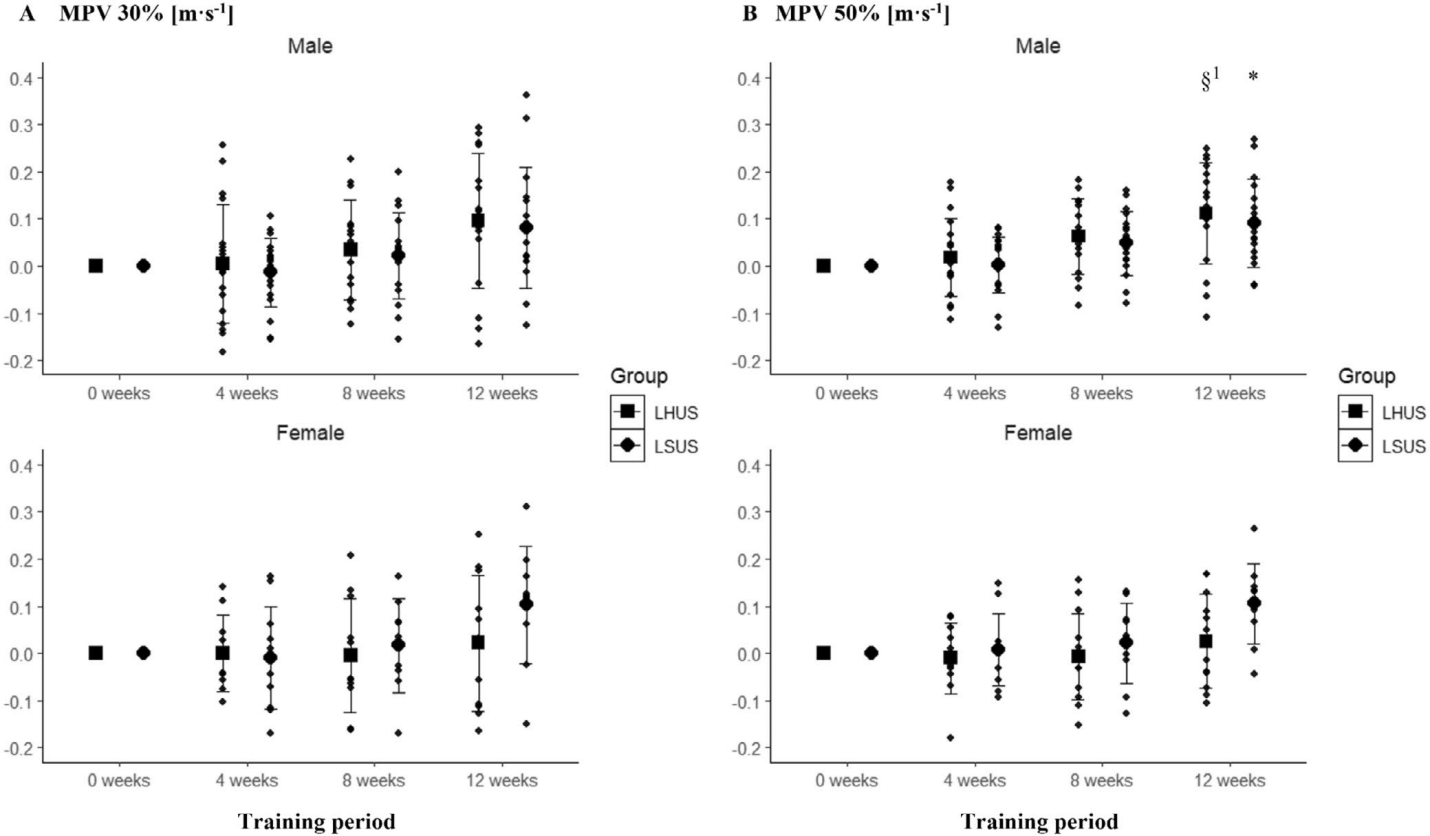
(A) Absolute change in MPV in the bench press for LHUS and LSUS over the 12‐week training period at 30% of the Week‐0 1RM clustered by sex. (B) Absolute change in MPV in bench press for LHUS and LSUS over the 12‐week training period at 50% of the Week‐0 1RM clustered by sex. *Indicates significant changes compared to baseline in LSUS. ^§1^Indicates significant changes compared to Week 4 in LHUS. LHUS, lower‐body high‐intensity interval training and upper‐body strength; LSUS, lower‐body and upper‐body strength; MPV, mean propulsive velocity.

The corresponding mean changes and standard deviations of MPVs are provided in Table S3 of Data S1. At 30% of 1RM, a significant main effect for time was observed in males (*p* = 0.006) and females (*p* = 0.036), but no significant interaction between time and group was found in either sex (males: *p* = 0.854, females: *p* = 0.109). However, pairwise comparison indicated no statistically significant differences between preintervention and postintervention (*p* > 0.050) (Figure 4A). At 50% of 1RM, a significant main effect for time was found in males (*p* < 0.001) and females (*p* = 0.001). Pairwise comparison indicated differences between preintervention and postintervention for LHUS (10.7% ± 16.9%; *p* = 0.003) and only between Week 4 and postintervention for LSUS (*p* = 0.009) in males but not in females (Figure 4B). No statistically significant interaction between time and group was observed for males (*p* = 0.884) but in females (*p* = 0.032). However, no between‐group differences were present at any time point (all, *p* = 1.000).

**FIGURE 5:**
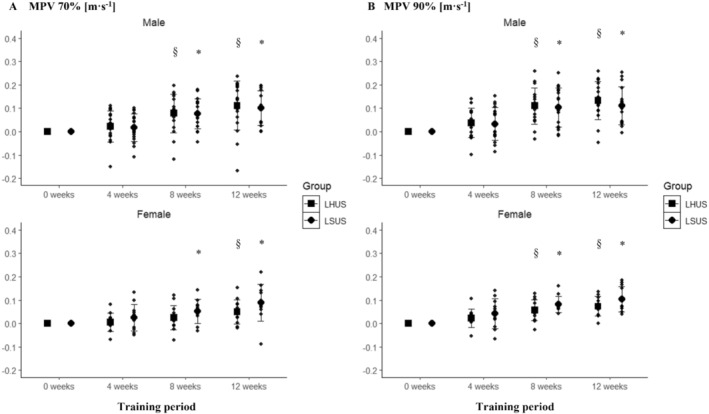
(A) Absolute change in MPV in the bench press for LHUS and LSUS over the 12‐week training period at 70% of the Week‐0 1RM clustered by sex. (B) Absolute change in MPV in bench press for LHLS and LSUS over the 12‐week training period at 90% of the Week‐0 1RM clustered by sex. *Indicates significant changes compared to baseline in LSUS. ^§^Indicates significant changes compared to baseline in LHUS. LHUS, lower‐body high‐intensity interval training and upper‐body strength; LSUS, lower‐body and upper‐body strength; MPV, mean propulsive velocity.

A statistically significant main effect was found at 70% of 1RM for time in males (*p* < 0.001) and females (*p* < 0.001), with no significant interaction between time and group for males (*p* = 0.768) but in females (*p* = 0.011). Pairwise comparisons showed statistically significant differences between preintervention and postintervention in LHUS for males (18.3% ± 17.1%; *p* = 0.002) and in females (11.0% ± 11.1%, *p* = 0.022), and statistically significant differences between preintervention and postintervention in LSUS for males (11.0% ± 11.1%; *p* < 0.001) and females (19.6% ± 19.3%; *p* = 0.012) (Figure 5A). At 90% of 1RM, a significant main effect was observed for time in males (*p* < 0.001) and females (*p* < 0.001), with no significant interaction between time and group for males (*p* = 0.792) or females (*p* = 0.081). Pairwise comparisons indicated statistically significant differences between preintervention and postintervention in LHUS (males: 38.6% ± 22.5%; *p* < 0.001; females: 26.5% ± 17.3%; *p* = 0.001) and LSUS (males: 34.2% ± 24.9%; *p* < 0.001; females: 38.5% ± 23.0%; *p* = 0.001) (Figure 5B).

### 3.2 Maximal Strength

#### 3.2.1 Same Muscle‐Group Concurrent Aerobic and Strength Training

**FIGURE 6:**
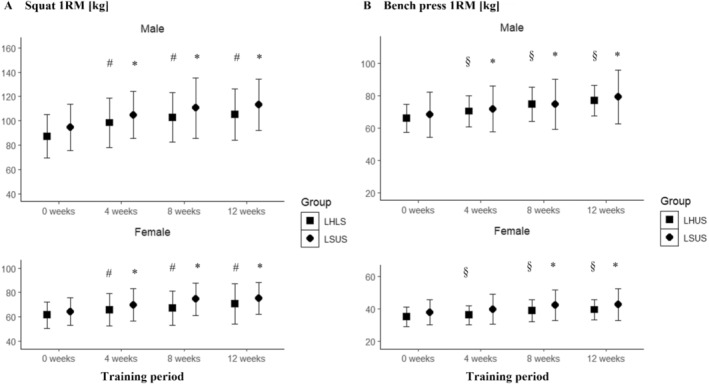
(A) 1RM in the squat for LHLS and LSUS over the 12‐week training period clustered by sex. (B) 1RM in the bench press for LHUS and LSUS over the 12‐week training period clustered by sex. *Indicates significant changes compared to baseline in LSUS. ^#^Indicates significant changes compared to baseline in LHLS. ^§^Indicates significant changes compared to baseline in LHUS. 1RM, one‐repetition maximum; HIIT, high‐intensity interval training; LHLS, lower‐body HIIT and lower‐body strength; LHUS, lower‐body HIIT and upper‐body strength; LSUS, lower‐body and upper‐body strength.

Significant main effects for time were observed in LHLS and LSUS in both males (19.3% ± 10.4% and 20.3% ± 10.4%, respectively; both groups: *p* < 0.001) and females (22.1% ± 11.9% and 17.4% ± 7.5%, respectively; both groups: *p* < 0.006). However, no significant interaction between time and group was found in males (*p* = 0.229) and females (*p* = 0.289) (Figure 6A).

#### 3.2.2 Separate Muscle‐Group Concurrent Aerobic and Strength Training

Significant main effects for time were found in LHUS and LSUS for males (17.3% ± 5.8% and 16.7% ± 14.0%, respectively; both groups: *p* < 0.001) and females (13.2% ± 8.7% and 12.8% ± 9.6%, respectively; both groups: *p* < 0.001), with no significant interaction between time and group in males (*p* = 0.654) and females (*p* = 0.714) (Figure 6B).

### 3.3 Muscle Cross‐Sectional Area

#### 3.3.1 Same Muscle‐Group Concurrent Aerobic and Strength Training

**FIGURE 7:**
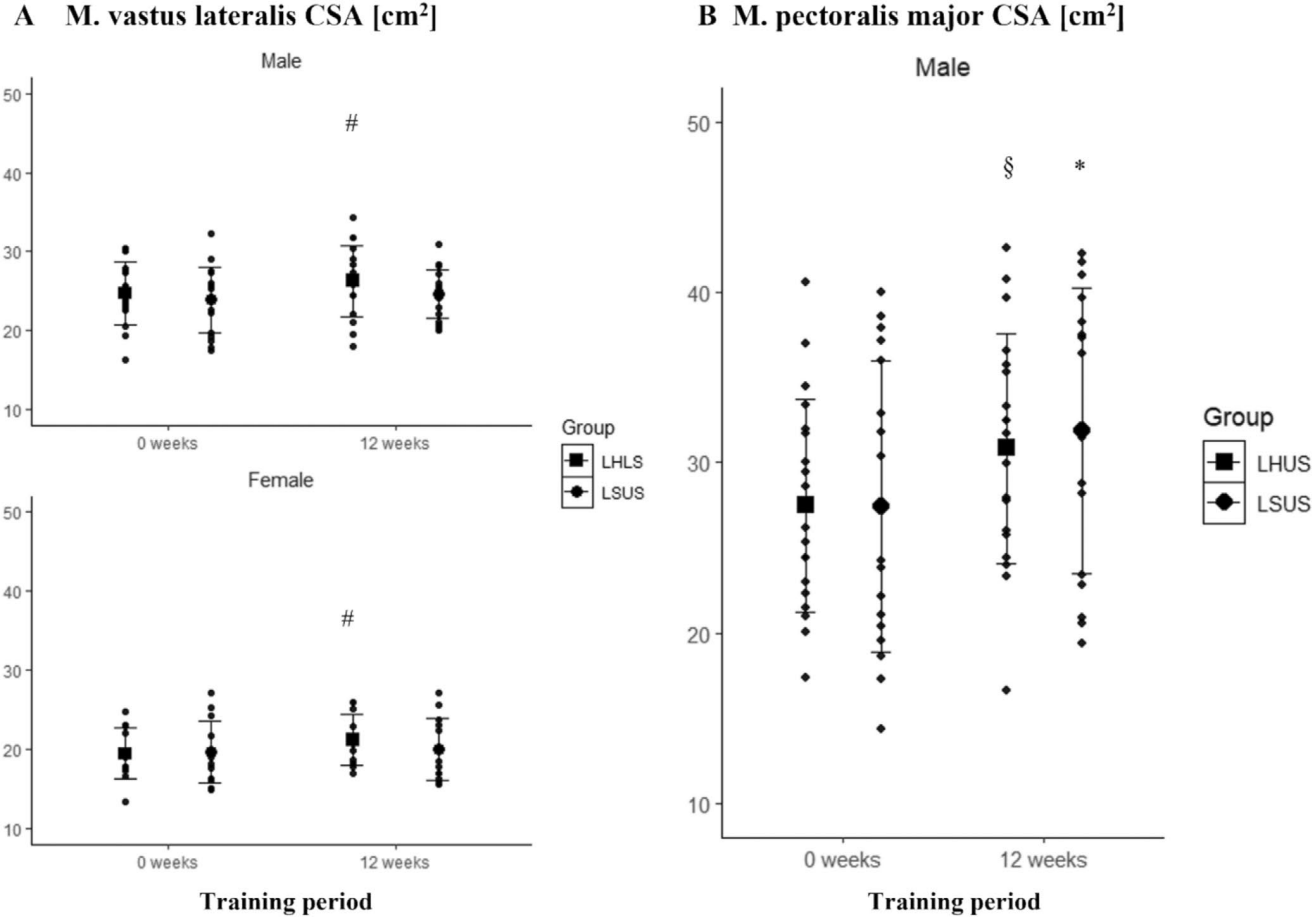
(A) M. vastus lateralis CSA in LHLS and LSUS over the 12‐week training period clustered by sex. (B) M. pectoralis major CSA in LHUS and LSUS over the 12‐week training period. *Indicates significant changes compared to baseline in LSUS. ^#^Indicates significant changes compared to baseline in LHLS. ^§^Indicates significant changes compared to baseline in LHUS. CSA, cross‐sectional area; HIIT, high‐intensity interval training; LHLS, lower‐body HIIT and lower‐body strength; LHUS, lower‐body HIIT and upper‐body strength; LSUS, lower‐body and upper‐body strength.

A significant main effect for time in M vastus lateralis CSA was found in males (*p* = 0.029) and females (*p* = 0.033), while no statistically significant interaction between time and group was observed for either males (*p* = 0.425) or females (*p* = 0.144). Pairwise comparisons showed a statistically significant increase in M. vastus lateralis CSA only for the LHLS group (males: 6.9% ± 10.0%; *p* = 0.016; females: 9.7% ± 11.1%; *p* = 0.031) (Figure 7A).

#### 3.3.2 Separate Muscle‐Group Concurrent Aerobic and Strength Training

A significant main effect for time in M. pectoralis major CSA was observed in males (*p* < 0.001), but no significant interaction between time and group was found (*p* = 0.636). Pairwise comparisons indicated statistically significant increases in M. pectoralis major CSA for both LHUS (13.2% ± 16.0%; *p* = 0.003) and LSUS (16.8% ± 15.8%; *p* < 0.001) (Figure 7B).

## 4 Discussion

The present study aimed to assess the effects of same muscle‐group (i.e., LHLS) versus separate muscle‐group (i.e., LHUS) concurrent aerobic and strength training on muscle function (i.e., explosive and maximal strength) and hypertrophy in recreationally active males and females. We found that both explosive and maximal strength improved to a similar extent irrespective of whether aerobic and strength training were performed within the same or in separate muscle groups compared to sole strength training. Additionally, we showed no statistically significant increases in M. vastus lateralis hypertrophy in LSUS but in LHLS, while M. pectoralis hypertrophy increased statistically significant to a similar extent in LSUS and LHUS. Moreover, no sex‐specific differences between males and females were found.

Over the 12‐week training period, we found that both males and females in the LHLS and LSUS groups showed significant improvements in MPVs at higher loads of 70% and 90% of 1RM for the squat exercise, while no significant improvements were observed at lower loads (i.e., 30% and 50% of 1RM). Similarly, for the bench press exercise, LHUS and LSUS showed significant increases in MPVs at 70% and 90% of 1RM, while no substantial changes were observed at 30% and 50% of 1RM. This improvement in MPVs that was only present at high loads may be attributed to the fact that much of the strength training was performed with similarly high loads (75%–90% 1RM), as enhancements in velocity at high loads often result from maximal strength training [27]. This observation is supported by previous research, which found that training at 15% of 1RM increased velocities at lower loads, while training at 90% of 1RM tended to improve velocity at higher loads [28]. This also aligns with studies assessing the knee extensor torque–velocity relationship, which also demonstrated that improvements in velocity are more likely to occur at higher loads and lower velocities, as the potential for adaptation at lower velocities may be greater. However, it is important to outline that despite large increases in squat MPV (70% 1RM: ~14% in males and ~12% in females; 90% 1RM: ~27% in males and ~19% in females) and bench press MPV (70% 1RM: ~17% in males and ~15% in females; 90% 1RM: ~36% in males and ~33% in females) high standard deviations were observed, indicating a somewhat heterogeneous response among participants.

Importantly, however, no significant interactions between time and group were found for LHLS versus LSUS in the squat and LHUS versus LSUS in the bench press, indicating that explosive strength increased to a similar extent irrespective of whether aerobic and strength training are performed within the same or in separate muscle groups compared to strength training only. Interestingly, females in the LSUS group showed significant improvements in squat MPV compared to baseline, whereas such changes were not observed for LHLS. However, since no statistical between‐group differences were present, it remains unclear whether this reflects interference in explosive strength. This is to some extent surprising. Based on our previous meta‐analysis [4], we hypothesized that an interference effect and thus an attenuated development in explosive strength would be apparent when aerobic and strength training are performed within the same muscle group. However, it is noteworthy that in this analysis, despite effect sizes indicating attenuated improvements [4], actual significant differences between strength training alone and combined aerobic and strength training were demonstrated in only six [2, 3, 9, 29, 30, 31] out of the 18 included studies. Thus, the absence of an interference effect in our research, particularly when comparing between‐group differences, aligns with previous findings, which also reported no interference effect on explosive strength [32, 33]. This discrepancy in findings may have likely been caused by differences in study designs, including variations in training characteristics such as training volume and frequency, as well as type of strength training. For example, two of the six aforementioned studies showed reduced adaptations in the countermovement jump (CMJ) performance when explosive strength training was immediately followed by running [3] or cycling [2]. In contrast to our study, which was periodized in 3 blocks: (1) hypertrophy, (2) maximal strength, and (3) explosive strength, we observed no maladaptation in explosive strength. Heterogeneity in results may also arise from different methods used to assess explosive strength, as demonstrated in a study where low‐intensity running following lower‐body power training led to comparable CMJ performance but impaired RFD [3].

Our findings suggest that separating aerobic and strength training by muscle group does not lead to interference in explosive strength development. However, our initial hypothesis, that aerobic and strength training would interfere with explosive strength development when performed within the same muscle group but not when targeting different muscle groups, was not fully supported. Nonetheless, this result is significant as it challenges the assumption of a generic interference effect on explosive strength, suggesting that this interference may not be attributable to specific exercise characteristics alone. For instance, research has indicated that longer aerobic sessions often lead to interference [5], while in fact, shorter HIIT intervals have shown interference in explosive strength as well [2, 3]. Moreover, it has been argued that the potential for interference may be more pronounced when aerobic and strength training are conducted within the same session [4]. However, some studies have also shown that training in aerobic endurance and strength on different days leads to interference in explosive strength [1], while in the present study same‐session concurrent aerobic and strength training did not lead to blunted explosive strength adaptations.

One potential way to better understand the interference effect may be to examine the underlying mechanisms at play. For instance, neural mechanisms have been proposed as possible origins of compromised neuromuscular adaptations following combined aerobic and strength training. Still, to date, only one study has truly focused on this [1]. They showed that the increase in integrated electromyographic signal during the first 500 ms of contraction was attenuated during combined as compared to single‐mode strength training, despite maximal voluntary activation showing no differences between the groups. Given the abundance of factors (e.g., neural activation, muscle morphology, and tendon properties) that may affect the explosive strength and thus possibly influence the occurrence of interference, future studies should consider these aspects to understand the mechanisms behind potential maladaptation.

Alongside changes in explosive strength, the 12‐week training period resulted in increases in 1RM strength for both the squat and bench press exercises. In the squat, LHLS and LSUS increased 1RM by approximately 19%–20% in males and 17%–22% in females. Similarly, the bench press 1RM in LHUS and LSUS saw increases of about 16%–17% in males and 13% in females. These improvements align with the magnitude of adaptations observed in previous studies for lower‐body strength gains following concurrent aerobic and strength training [1, 29]. However, while earlier research has indicated that maximal strength is generally unaffected by concurrent aerobic and strength training when targeting the same muscle group [4, 5], there has been limited evidence of how combining lower‐body aerobic exercise with upper‐body strength training impacts upper‐body maximal strength. Notably, it was previously demonstrated that upper‐body maximal strength improves similarly whether upper‐body strength training is performed alone or combined with lower‐body aerobic exercise as well [6], aligning with findings from our study.

Interestingly, we found increases in CSA for the M. vastus lateralis, where the increases in the LHLS group were larger compared to the LSUS group (6.9% and 5.1% in males, 9.7% and 2.4% in females, respectively), as only the LHLS group showed statistically significant improvements. In contrast, the LHUS and LSUS groups increased M. pectoralis major CSA to a similar extent (13.2% and 16.8%, respectively), indicating that aerobic training did not affect hypertrophy in the upper body. These findings align with previous research suggesting that combining aerobic and strength training may actually enhance muscle growth compared to strength training alone, indicating that aerobic exercise may provide an additional hypertrophic stimulus [34]. However, this effect may depend on the type of aerobic exercise, as running has not been shown to promote muscle hypertrophy, whereas cycling, in turn, has been found to induce increases in muscle mass [1, 35], which challenges the previously proposed generic molecular interference mechanism [36]. This mechanism suggests that aerobic exercise activates AMP‐activated protein kinase (AMPK), which inhibits mTOR signaling and thereby limits muscle growth [37].

It is somewhat surprising, however, that strength training alone did not induce statistically significant improvements in the M. vastus lateralis CSA for both males and females (5.1% ± 15.8% and 2.4% ± 11.3%, respectively) despite leading to significant increases in M. pectoralis major CSA in males. Since we included recreationally active individuals, muscle hypertrophy of the lower body may be limited since these muscle groups are already engaged in daily activities. Thus, the potential for additional adaptation from training may be less pronounced. This suggests that, at least for M. vastus lateralis CSA, the current training regimen, which dedicated only 4 weeks to hypertrophy‐focused training, may not have provided a sufficient stimulus for significant muscle growth in recreationally active individuals.

Although our study adds to the current evidence about concurrent strength and aerobic training, limitations should be acknowledged. First, no markers of neural activation, such as voluntary activation, were included. Second, the observation of no interference in recreationally active individuals is an intriguing finding; however, it may have limited applicability to other populations, particularly those with a systematic training history. As such, these results should be considered population‐specific and interpreted with caution when extrapolating to highly trained or elite populations. Finally, CSA measurements of the M. vastus lateralis were used, which may not fully capture changes in muscle mass at the whole‐quadriceps level compared to methods such as magnetic resonance imaging.

### 4.1 Perspectives

The present study demonstrated that concurrent aerobic and strength training does not impair maximal or explosive strength development nor attenuates muscle CSA, irrespective of whether it is performed within the same or separate muscle groups. In addition, both males and females showed similar improvements in strength capacity and muscle CSA, with no evidence of a sex‐specific interference effect. These findings do not support a generic interference effect on explosive strength, suggesting that potential interference may be influenced by specific training characteristics, such as overall training volume and frequency, type of aerobic and strength training, and participants' training status. Further research is needed to explore the mechanisms behind these adaptations, particularly focusing on neural factors and the effects of muscle architecture and tendon properties on explosive strength.

## Ethics Statement

This study received approval from the local institutional board and adhered to the principles of the Declaration of Helsinki (German Sport University, 036/2019).

## Conflicts of Interest

The authors declare no conflicts of interest.

## Supporting information


Data S1

## Data Availability

Data are available upon reasonable request.
