# Supplementary material for: Neuromuscular Adaptations to Same Versus Separate Muscle‐Group Concurrent Aerobic and Strength Training in Recreationally Active Males and Females
Source: Scand J Med Sci Sports. 2025 Feb 8;35(2):e70025. doi: 10.1111/sms.70025 (PMC11806282; doi:10.1111/sms.70025)
Supplement: Supplementary file 1 — Data S1 [file SMS-35-e70025-s001.docx]

Table S1 Exercises for the lower- and upper body with the respective loads over the 12-week training intervention.

| Training period | | | Exercises (lower-body & upper-body) with respective loads | | | |
| --- | --- | --- | --- | --- | --- | --- |
| Block | Week | Session | Squat &  Bench press | Leg press &  Incline bench press | Leg curl &  Seated rowing | Leg extension &  Triceps extension |
| 1 | 1 | 1 | 3x10 75%RM | 3x10 75%RM | 3x10 75%RM | 3x10 75%RM |
|  |  | 2 | 3x8 80%RM | 3x8 80%RM | 3x8 80%RM | 3x8 80%RM |
|  | 2 | 1 | 3x10 75%RM | 3x10 75%RM | 3x10 75%RM | 3x10 75%RM |
|  |  | 2 | 3x8 80%RM | 3x8 80%RM | 3x8 80%RM | 3x8 80%RM |
|  | 3 | 1 | 3x10 75%RM | 3x10 75%RM | 3x10 75%RM | 3x10 75%RM |
|  |  | 2 | 3x8 80%RM | 3x8 80%RM | 3x8 80%RM | 3x8 80%RM |
|  | 4 | 1 | 2x5 85%RM | 2x5 85%RM | 2x5 85%RM | 2x5 85%RM |
|  |  | 2 | 1RM Test |  |  |  |
| 2 | 1 | 1 | 3x5 85%RM | 3x5 85%RM | 3x5 85%RM | 3x5 85%RM |
|  |  | 2 | 3x3 90%RM | 3x3 90%RM | 3x3 90%RM | 3x3 90%RM |
|  | 2 | 1 | 3x5 85%RM | 3x5 85%RM | 3x5 85%RM | 3x5 85%RM |
|  |  | 2 | 3x3 90%RM | 3x3 90%RM | 3x3 90%RM | 3x3 90%RM |
|  | 3 | 1 | 3x5 85%RM | 3x5 85%RM | 3x5 85%RM | 3x5 85%RM |
|  |  | 2 | 3x3 90%RM | 3x3 90%RM | 3x3 90%RM | 3x3 90%RM |
|  | 4 | 1 | 2x5 85%RM | 2x5 85%RM | 2x5 85%RM | 2x5 85%RM |
|  |  | 2 | 1RM Test |  |  |  |
| 3 | 1 | 1 | 3x6 40%RM | 3x6 40%RM | 3x5 85%RM | 3x5 85%RM |
|  |  | 2 | 3x3 90%RM | 3x3 90%RM | 3x3 90%RM | 3x3 90%RM |
|  | 2 | 1 | 3x6 40%RM | 3x6 40%RM | 3x5 85%RM | 3x5 85%RM |
|  |  | 2 | 3x6 40%RM | 3x6 40%RM | 3x5 85%RM | 3x5 85%RM |
|  | 3 | 1 | 3x6 40%RM | 3x6 40%RM | 3x5 85%RM | 3x5 85%RM |
|  |  | 2 | 3x6 40%RM | 3x6 40%RM | 3x5 85%RM | 3x5 85%RM |
|  | 4 | 1 | 3x3 90%RM | 3x3 90%RM | 3x3 90%RM | 3x3 90%RM |
|  |  | 2 | 3x5 85%RM | 3x5 85%RM | 3x5 85%RM | 3x5 85%RM |
|  |  | 3 | 2x5 85%RM | 2x5 85%RM | 2x5 85%RM | 2x5 85%RM |

Table S2 Changes in squat MPV at pre-test loads for males and females. LSUS = lower-body and upper-body strength, LHLS = lower-body HIT and lower-body strength, MPV= mean propulsive velocity.

| **Time**  **[weeks]** | **Group** | **Sex** | Δ MPV 30%  [m·s^-1^] | Δ MPV 50%  [m·s^-1^] | Δ MPV 70%  [m·s^-1^] | Δ MPV 90%  [m·s^-1^] |
| --- | --- | --- | --- | --- | --- | --- |
| **0** | **LHLS** | **male** | 0 ± 0 | 0 ± 0 | 0 ± 0 | 0 ± 0 |
| **4** |  |  | 0.007 ± 0.111 | 0.031 ± 0.086 | 0.056 ± 0.071 | 0.080 ± 0.072 |
| **8** |  |  | -0.002 ± 0.091 | 0.034 ± 0.075 | 0.071 ± 0.067 | 0.108 ± 0.068 |
| **12** |  |  | 0.033 ± 0.094 | 0.058 ± 0.076 | 0.082 ± 0.067 | 0.106 ± 0.070 |
| **0** | **LSUS** | **male** | 0 ± 0 | 0 ± 0 | 0 ± 0 | 0 ± 0 |
| **4** |  |  | 0.001 ± 0.088 | 0.024 ± 0.075 | 0.046 ± 0.067 | 0.069 ± 0.066 |
| **8** |  |  | 0.019 ± 0.133 | 0.051 ± 0.103 | 0.082 ± 0.078 | 0.114 ± 0.065 |
| **12** |  |  | 0.043 ± 0.091 | 0.068 ± 0.079 | 0.094 ± 0.072 | 0.120 ± 0.072 |
| **0** | **LHLS** | **female** | 0 ± 0 | 0 ± 0 | 0 ± 0 | 0 ± 0 |
| **4** |  |  | -0.014 ± 0.102 | 0.003 ± 0.066 | 0.021 ± 0.038 | 0.039 ± 0.041 |
| **8** |  |  | -0.049 ± 0.087 | -0.025 ± 0.059 | -0.001 ± 0.034 | 0.024 ± 0.026 |
| **12** |  |  | 0.012 ± 0.115 | 0.03 ± 0.085 | 0.048 ± 0.064 | 0.066 ± 0.060 |
| **0** | **LSUS** | **female** | 0 ± 0 | 0 ± 0 | 0 ± 0 | 0 ± 0 |
| **4** |  |  | 0.021 ± 0.091 | 0.033 ± 0.062 | 0.044 ± 0.043 | 0.056 ± 0.049 |
| **8** |  |  | 0.059 ± 0.094 | 0.065 ± 0.063 | 0.071 ± 0.040 | 0.076 ± 0.042 |
| **12** |  |  | 0.045 ± 0.113 | 0.053 ± 0.078 | 0.062 ± 0.055 | 0.071 ± 0.060 |

Table S3. Changes in bench press MPV at pre-test loads for males and females. LSUS = lower-body and upper-body strength, LHUS = lower-body HIT and upper-body strength, MPV= mean propulsive velocity.

| **Time**  **[weeks]** | **Group** | **Sex** | Δ MPV 30%  [m·s^-1^] | Δ MPV 50%  [m·s^-1^] | Δ MPV 70%  [m·s^-1^] | Δ MPV 90%  [m·s^-1^] |
| --- | --- | --- | --- | --- | --- | --- |
| **0** | **LHUS** | **male** | 0 ± 0 | 0 ± 0 | 0 ± 0 | 0 ± 0 |
| **4** |  |  | -0.009 ± 0.136 | 0.007 ± 0.096 | 0.023 ± 0.066 | 0.038 ± 0.062 |
| **8** |  |  | 0.015 ± 0.131 | 0.046 ± 0.103 | 0.078 ± 0.083 | 0.11 ± 0.078 |
| **12** |  |  | 0.068 ± 0.182 | 0.09 ± 0.141 | 0.111 ± 0.105 | 0.133 ± 0.081 |
| **0** | **LSUS** | **male** | 0 ± 0 | 0 ± 0 | 0 ± 0 | 0 ± 0 |
| **4** |  |  | -0.013 ± 0.073 | 0.003 ± 0.06 | 0.018 ± 0.059 | 0.033 ± 0.07 |
| **8** |  |  | 0.022 ± 0.091 | 0.049 ± 0.068 | 0.076 ± 0.065 | 0.103 ± 0.084 |
| **12** |  |  | 0.082 ± 0.128 | 0.091 ± 0.094 | 0.101 ± 0.075 | 0.111 ± 0.081 |
| **0** | **LHUS** | **female** | 0 ± 0 | 0 ± 0 | 0 ± 0 | 0 ± 0 |
| **4** |  |  | -0.022 ± 0.112 | -0.005 ± 0.073 | 0.012 ± 0.042 | 0.029 ± 0.043 |
| **8** |  |  | -0.039 ± 0.133 | -0.005 ± 0.089 | 0.029 ± 0.052 | 0.063 ± 0.047 |
| **12** |  |  | 0.008 ± 0.148 | 0.031 ± 0.097 | 0.053 ± 0.051 | 0.075 ± 0.041 |
| **0** | **LSUS** | **female** | 0 ± 0 | 0 ± 0 | 0 ± 0 | 0 ± 0 |
| **4** |  |  | 0.004 ± 0.113 | 0.017 ± 0.08 | 0.031 ± 0.058 | 0.044 ± 0.061 |
| **8** |  |  | 0.010 ± 0.128 | 0.041 ± 0.103 | 0.073 ± 0.087 | 0.105 ± 0.086 |
| **12** |  |  | 0.075 ± 0.176 | 0.090 ± 0.129 | 0.105 ± 0.092 | 0.120 ± 0.077 |

Results

Maximal Strength

Same muscle group concurrent aerobic and strength training

LHLS and LSUS increased squat 1RM over the 12 weeks by 19.3% ± 10.4% and 20.3% ± 10.4% in males and 22.1% ± 11.9% and 17.4% ± 7.5% in females. Significant main effects were observed for time in males (F(3, 90) = 84.7, p < 0.001, η² = 0.738) and females (F(1.88,) = 45.2, p < 0.001, η² = 0.634 ). However, there was no significant interaction between time and group in males (F(3, 90) = 1.47, p = 0.229, η² = 0.047) and females (F(1.88, 41.43) = 1.28, p = 0.289, η² = 0.289). In the LHLS group, significant differences were observed between time points 1 and 4 for males (t = -8.00, df = 15, p < 0.001) and females (t = -4.32, df = 12, p = 0.006). For the LSUS group, significant differences were found between time points 1 and 4 (t = -9.98, df = 19, p < 0.001) in males and females (t = -7.83, df = 12, p < 0.001).

Different muscle groups concurrent aerobic and strength training

LHUS and LSUS increased bench press 1RM over the 12 weeks by 17.3% ± 5.8% and 16.7% ±14.0% in males and 13.2% ± 8.7% and 12.8% ± 9.6% in females. Significant main effects were observed for time in males (F(1.96,54.9) = 47.2, p < 0.001, η² = 0.628) and females (F(1.97, 43.2) = 30.9, p < 0.001, η² = 0.584). However, there was no significant interaction between time and group (F(1.96,54.9) = 0.422, p = 0.654, η² = 0.015 for males; F(1.97, 43.2) = 0.456, p = 0.714, η² = 0.020 for females). In the LHUS group, significant differences were observed between time points 1 and 4 for males (t = -12.3, df = 16, p < 0.001) and females (t = -5.30, df = 13, p = 0.001). Similarly, for the LSUS group, significant differences were found between time points 1 and 4 for males (t = -5.57, df = 18, p < 0.001) and females (t = -4.57, df = 12, p = 0.003).

Explosive strength

Same muscle group concurrent aerobic and strength training

LHLS and LSUS increased squat MPV at 30% over the 12 weeks by 3.7% ± 10.0% and 4.5% ± 8.9% in males and 2.5% ± 12.3% and 11.7% ± 26.3% in females. LHLS and LSUS increased squat MPV at 50% over the 12 weeks by 7.5% ± 10.1% and 8.5% ± 9.5% in males and 5.3% ± 11.3% and 12.6% ± 21.2% in females. No significant main effects were observed for time (F(2.16, 47.4) = 47.4, p = 0.187, η² = 0.073 for males; F(3, 45) = 0.797, p = 0.502, η² = 0.050 for females) nor the interaction between time and group (F(2.16, 47.4) = 0.257, p = 0.790, η² = 0.012 for males; F(3, 45) = 1.06, p = 0.374, η² = 0.066 for females) for MPVs at 30% of 1RM. For MPVs at 50% of 1RM, a significant main effect was found for time in males (F(2.13, 47.0) = 4.13, p = 0.020, η² = 0.158) but not in females (F(3, 45) = 1.97, p = 0.132, η² = 0.116). Pairwise comparison indicated no significant differences between time points 1 and 4 in both the LHLS (p = 0.067) and LSUS (p = 0.054) groups. Additionally, there was no significant interaction between group and time in males (F(2.13, 47.0) = 0.255, p = 0.790, η² = 0.011) and females (F(3, 45) = 1.44, p = 0.244, η² = 0.088).

LHLS and LSUS increased squat MPV at 70% over the 12 weeks by 13.7% ± 11.8% and 15.3% ± 11.7% in males and 9.7% ± 11.9% and 14.7% ± 15.7% in females. LHLS and LSUS increased squat MPV at 90% over the 12 weeks by 26.2% ± 19.6% and 28.9% ± 18.2% in males and 18.1% ± 18.6% and 20.3% ± 17.3% in females. For MPVs at 70% a significant main effect was observed for time in males (F(2.19, 48.3) = 13.8, p < 0.001, η² = 0.385) and females (F(3, 45) = 7.87, p < 0.001, η² = 0.344). However, neither males (F(2.19, 48.3) = 0.292, p = 0.768, η² = 0.013) nor females (F(3, 45) = 2.37, p = 0.083, η² = 0.137) showed significant interactions between group and time. Pairwise comparisons indicated that in the LHLS group, significant differences were observed between time points 1 and 4 for males (t = -8.00, df = 15, p < 0.001) but not in females (t = -2.72, df = 12, p = 0.112). For the LSUS group, significant differences were found between time points 1 and 4 for males (t = -9.98, df = 19, p < 0.001) and females (t = -3.64, df = 11, p = 0.023). For MPVs at 90% 1RM, a significant main effect of time was observed for males (F(2.34, 51.4) = 27.9, p < 0.001, η² = 0.559) and females (F(3, 45) = 24.0, p < 0.001, η² = 0.616). For the interaction effects, a significant interaction between group and time was found in females (F(3, 45) = 3.46, p = 0.024, η² = 0.187), whereas no significant interaction was observed in males (F(2.34, 51.4) = 0.366, p = 0.728, η² = 0.016). Pairwise comparison indicated significant differences between time points 1 and 4 in the LHLS in males (t = -8.00, df = 15, p < 0.001) and females (t = -4.01, df = 12, p = 0.010). Similarly, in LSUS significant differences were also observed between time points 1 and 4 in males (t = -9.98, df = 19, p < 0.001) and females (t = -4.09, df = 11, p = 0.011). Pairwise comparison showed no statistically significant difference between the groups (p > 0.050).

Different muscle group concurrent aerobic and strength training

LHUS and LSUS increased bench press MPV at 30% over the 12 weeks by 6.7% ± 17.4% and 7.7% ± 12.1% in males and 2.7% ± 15.7% and 9.0% ± 22.1% in females. LHUS and LSUS increased bench press MPV at 50% over the 12 weeks by 10.7% ± 16.9% and 10.9% ± 11.8% in males and 6.4% ± 14.0% and 12.7% ± 20.8% in females. The MPV at 30 and 50% for the bench press, grouped by sex, for the time points are visualized in Figure 4. At 30% of the 1RM, a significant main effect was observed for time in males (F(2.13, 55.4) = 5.38, p = 0.006, η² = 0.171) and in females (F(3, 51) = 3.07, p = 0.036, η² = 0.153). However, no interaction between time and group was present for males (F(2.13, 55.4) = 0.174, p = 0.854, η² = 0.007) and females (F(3, 51) = 2.12, p = 0.109, η² = 0.111). Pairwise comparison indicated no statistically significant differences between time points 1 and 4 for either the LHUS or the LSUS group.

At 50% of the 1RM, a significant main effect was observed for time in males (F(2.02, 52.6) = 12.2, p < 0.001, η² = 0.319) and in females (F(3,51) = 7.10, p = 0.001, η² = 0.295). Pairwise comparison indicated statistically significant differences between time points 1 and 4 for LHUS (t = -4.29, df = 17, p = 0.003) and between time points 2 and 4 for LSUS (t = -3.87, df= 15, p= 0.009) in males but not in females. However, no interaction between time and group was present for males (F(2.02, 52.6) = 0.126, p = 0.884, η² = 0.007) but in females (F(3, 51) = 3.17, p = 0.032, η² = 0.157). No between-group differences were present at any time points (all, p =1.000).

LHUS and LSUS increased bench press MPV at 70% over the 12 weeks by 18.3% ± 17.1% and 17.0% ± 13.3% in males and 11.0% ± 11.1% and 19.6% ± 19.3% in females. LHUS and LSUS increased bench press MPV at 90% over the 12 weeks by 38.6% ± 22.5% and 34.2% ± 24.9% in males and 26.5% ± 17.3% and 38.5% ± 23.0% in females. At 70% of the 1RM, a significant main effect was observed for time in males (F(2.02, 52.6) = 28.6, p < 0.001, η² = 0.524) and in females (F(3,51) = 18.94, p < 0.001, η² = 0.527). However, no interaction between time and group was present for males (F(2.02, 52.6) = 0.269, p = 0.768, η² = 0.010) but for females (F(3, 51) = 4.14, p = 0.011, η² = 0.196). Pairwise comparisons indicated that in the LHUS group, significant differences were observed between time points 1 and 4 for males (t = -4.52, df = 17, p = 0.002) and in females (t = -3.66, df = 11, p = 0.022). For the LSUS group, significant differences were found between time points 1 and 4 for males (t = -9.98, df = 19, p < 0.001) and females (t = -4.04, df = 11, p = 0.012). No between-group differences were present at any time points (all, p =1.000).

At 90% of the 1RM, a significant main effect was observed for time in males (F(2.29, 59.4) = 59.4, p < 0.001, η² = 0.635) and in females (F(3,51) = 23.39, p < 0.001, η² = 0.579). However, no interaction between time and group was present for males (F(2.29, 59.4) = 0.792, p = 0.792, η² = 0.010) and females (F(3, 51) = 2.37, p = 0.081, η² = 0.122). Pairwise comparisons indicated that in the LHUS group, significant differences were observed between time points 1 and 4 for males (t = -6.4, df = 17, p < 0.001) and in females (t = -6.18, df = 11, p < 0.001). For the LSUS group, significant differences were found between time points 1 and 4 for males (t = -5.49, df = 15, p < 0.001) and females (t = -5.39, df = 11, p = 0.001). No between-group differences were present at any timepoints (all, p =1.000).

.

Muscle cross-sectional area

Same muscle group concurrent aerobic and strength training

LHLS and LSUS increased m. vastus lateralis CSA over the 12 weeks by 6.9% ± 10.0% and 5.1% ± 15.8% in males and 9.7% ± 11.1% and 2.4% ± 11.3% in females. For m. vastus lateralis CSA a significant main effect was observed for time in males (F(1, 33) = 5.23, p = 0.029, η² = 0.137) and in females (F(1, 21) = 5.22, p = 0.033, η² = 0.199). No interaction between time and group was present for males (F(1, 33) = 0.653, p = 0.425, η² = 0.019) and for females (F(1, 21) = 2.30, p = 0.144, η² = 0.099). Pairwise comparison indicated that in males and females only for LHLS, a significant increase was present (p = 0.016, p = 0.031, respectively). However, no between-group differences were present at baseline or following the 12-week intervention (p = 0.893 and p = 0.620, respectively).

Different muscle group concurrent aerobic and strength training

LHUS and LSUS increased m. pectoralis major CSA over the 12 weeks by 13.2% ± 16.0% and 16.8% ± 15.8% in males. For m. pectoralis major CSA, a significant main effect was observed for time in males (F(1, 33) = 30.8, p < 0.001, η² = 0.482), while no interaction between time and group was present (F(1, 33) = 0.228, p = 0.636, η² = 0.007). Pairwise comparison indicated that in males a significant increase was present for LHUS (p = 0.003) and LSUS (p < 0.001).


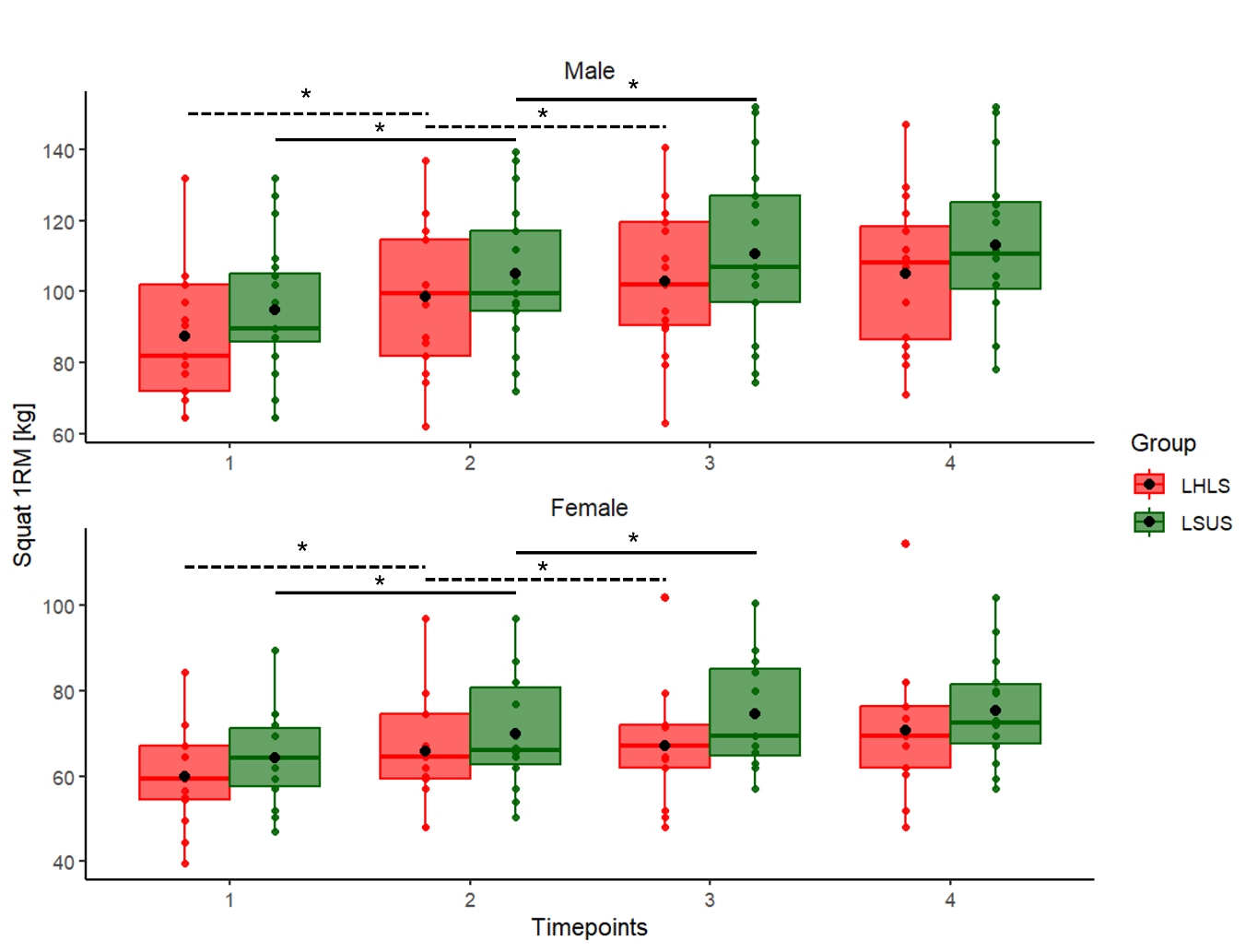


Figure 1. Squat one-repetition maximum (1RM) at time points 1-4 clustered sex. Solid black points represent the mean values. Asterisks (*) indicate statistically significant changes within the group over adjacent time points. The dotted lines represent comparisons for the LHLS group. Solid lines represent comparisons for the LSUS group.


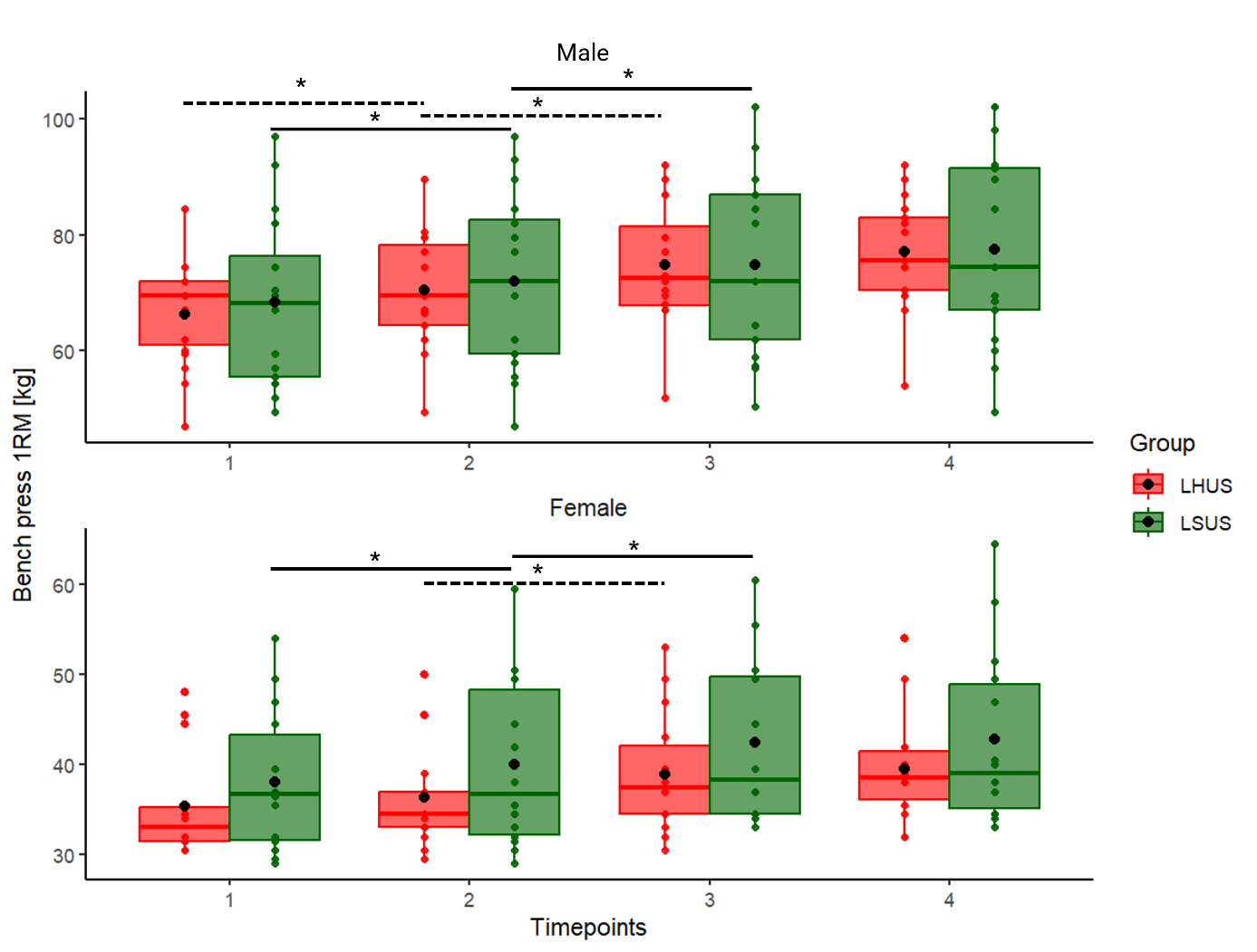


Figure 2. Bench press one-repetition maximum (1RM) at time points 1-4 clustered sex. Solid black points represent the mean value. Asterisks (*) indicate statistically significant changes within the group over adjacent time points. The dotted lines represent comparisons for the LHUS group. Solid lines represent comparisons for the LSUS group.


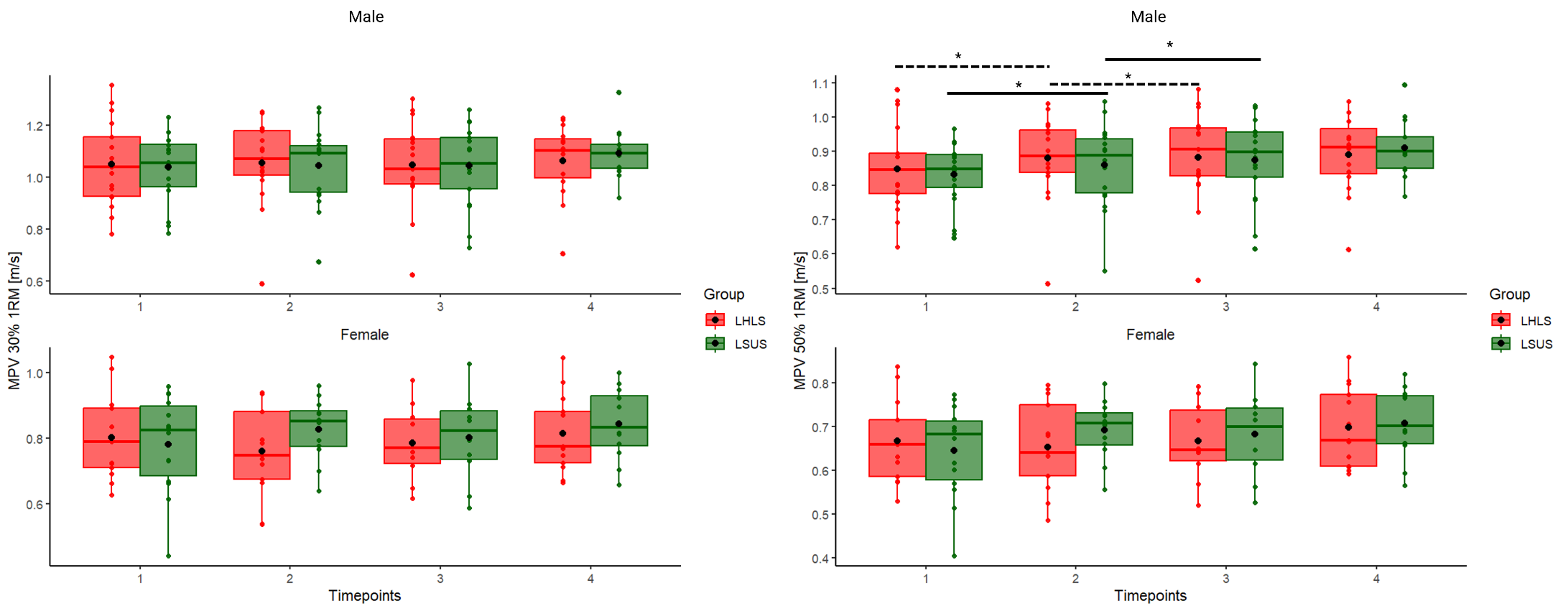


Figure 3. MPVs at 30% and 50% of 1RM at time points 1-4 clustered by sex. Solid black points represent the mean value. Asterisks (*) indicate statistically significant changes within the group over adjacent time points. The dotted lines represent comparisons for the LHLS group. Solid lines represent comparisons for the LSUS group.


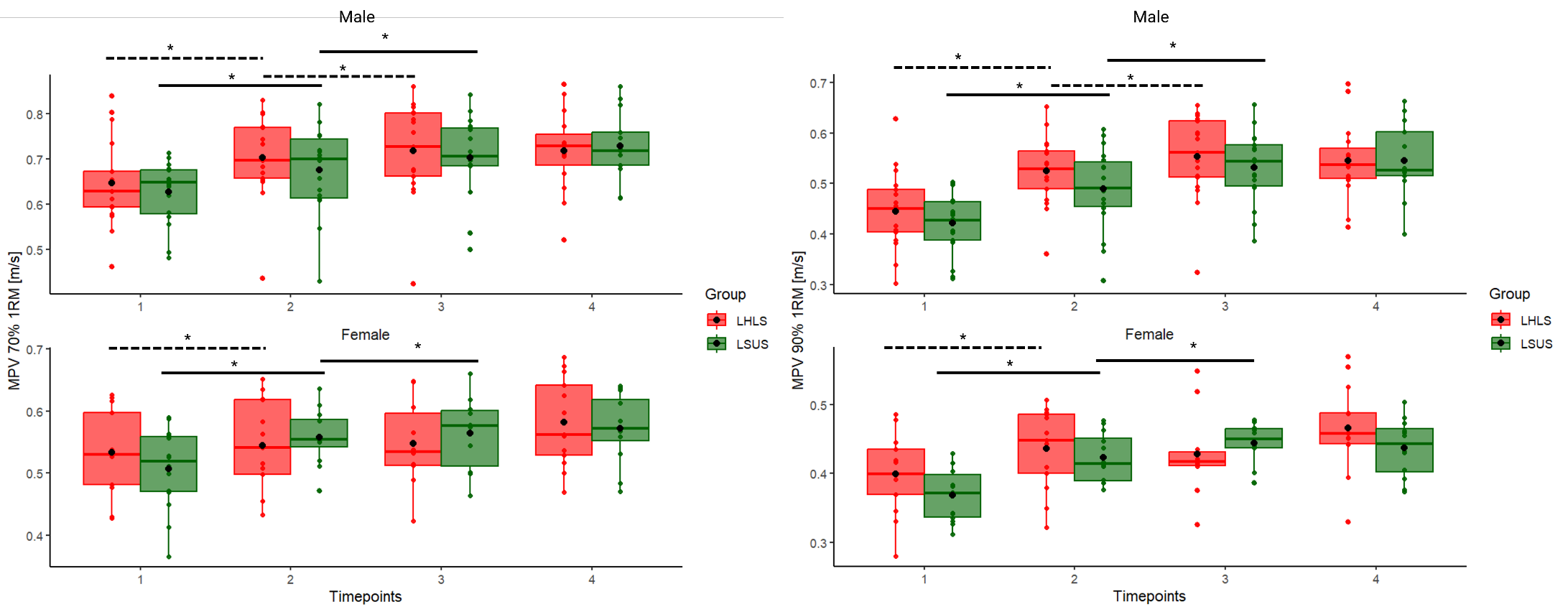


Figure 4. Squat MPVs at 70% and 90% of 1RM at time points 1-4 clustered by sex. Solid black points represent the mean value. Asterisks (*) indicate statistically significant changes within the group over adjacent time points. The dotted lines represent comparisons for the LHLS group. Solid lines represent comparisons for the LSUS group.


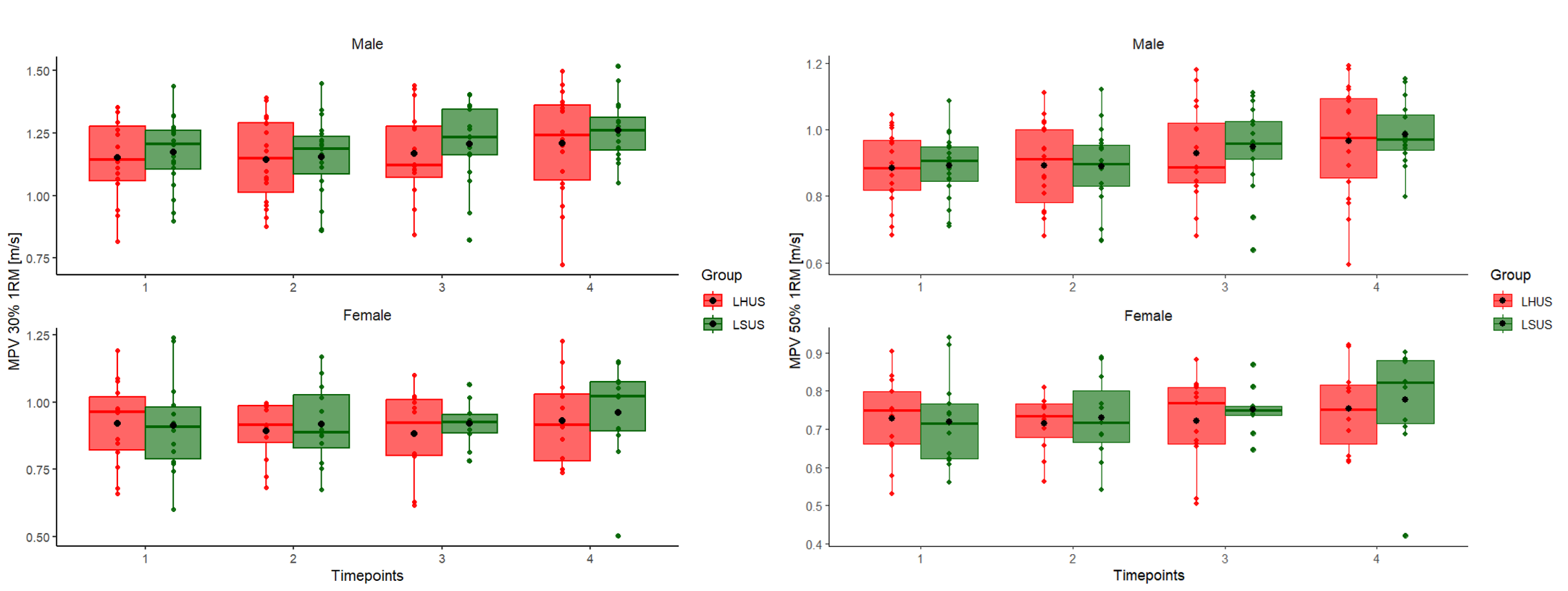


Figure 5. Bench press MPVs at 30% and 50% of 1RM at time points 1-4 clustered by sex. Solid black points represent the mean value


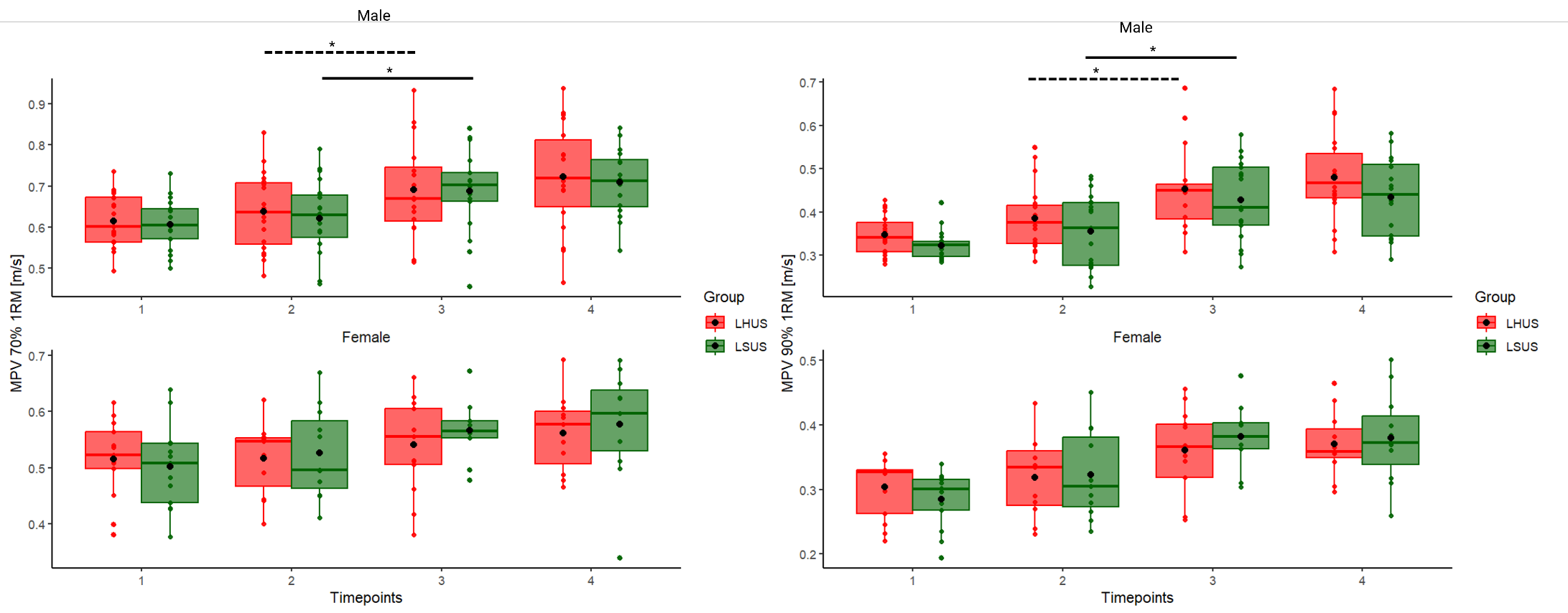


Figure 6. Bench press MPVs at 70% and 90% of 1RM at time points 1-4 clustered by sex. Solid black points represent the mean value. Asterisks (*) indicate statistically significant changes within the group over adjacent time points. The dotted lines represent comparisons for the LHUS group. Solid lines represent comparisons for the LSUS group.
